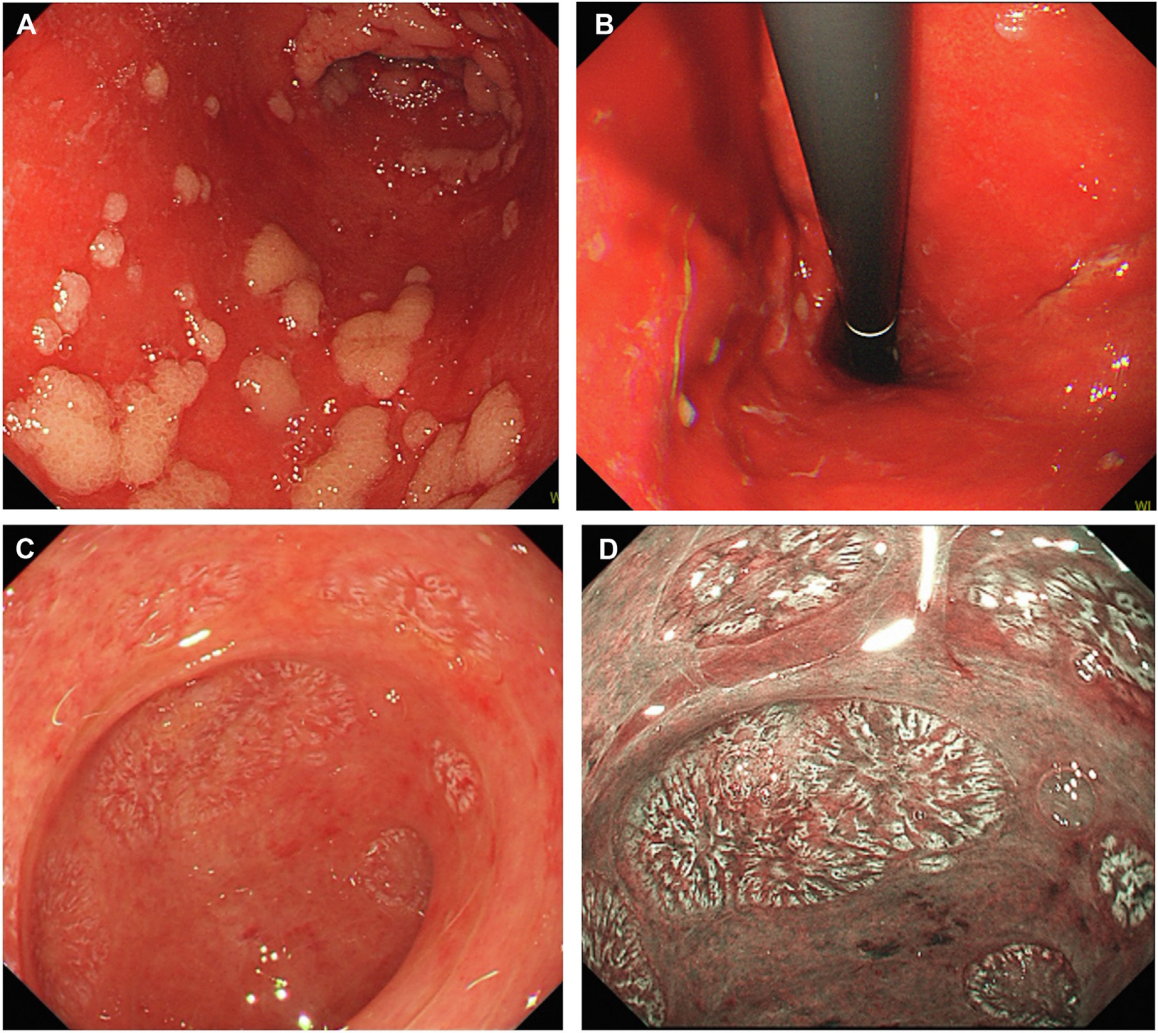# Diffuse Sloughing of Gastrointestinal Mucosa in Acute Upper Gastrointestinal Graft-Versus-Host Disease

**DOI:** 10.1016/j.gastha.2023.06.012

**Published:** 2023-07-05

**Authors:** Yasutoshi Shiratori, Katsuyuki Fukuda

**Affiliations:** Division of Gastroenterology, St. Luke’s International Hospital, Tokyo, Japan

A 72-year-old female presented with complaints of severe nausea, abdominal pain, and melena, 25 days after undergoing allogeneic hematopoietic stem cell transplantation for anaplastic large cell lymphoma. The patient reported no significant history of digestive difficulties. However, an esophagogastroduodenoscopy revealed a wide range of reddish ulcerations and edema of the remaining mucosa in the stomach (Figure A and B). Her duodenum exhibited extensive loss of villi (Figure C); and villous formations were clearly observed on narrow-band imaging (Figure D). A biopsy of the inflamed area in the stomach revealed crypt loss and apoptosis, and cytomegalovirus staining was negative. The patient was diagnosed with acute gastrointestinal graft-versus-host disease (GVHD), which prompted the immediate initiation of systemic prednisolone therapy. Thereafter, symptoms improved within 2 weeks. Organ transplantation is well established and performed with increasing frequency. GVHD often involves the skin, liver, and gastrointestinal tract. As this case demonstrates, although gastrointestinal symptoms in GVHD are nonspecific, massive mucosal loss is evident with endoscopy, providing a specific and useful indication for diagnosis. In addition, narrow band imaging may be superior to white-light imaging for assessing the shape of villi (loss, atrophy, and structure) in the duodenum.

**Figure:**